# Supplementary material for: The deubiquitinase (DUB) USP13 promotes Mcl-1 stabilisation in cervical cancer
Source: Oncogene. 2021 Feb 24;40(11):2112–29. doi: 10.1038/s41388-021-01679-8 (PMC7979541; doi:10.1038/s41388-021-01679-8)
Supplement: Supplementary file 1 — Supplementary Figure legends [file 41388_2021_1679_MOESM1_ESM.docx]

**Supplementary Figure 1. Copy Number Alterations of deubiquitinating enzymes in cervical cancer from the TCGA database.** **A)** Copy number amplifications of deubiquitinating enzymes in cervical cancer using a previously curated database (35). **B)** Copy number deletions of deubiquitinating enzymes in cervical cancer using a previously curated database (35).

**Supplementary Figure 2. *USP13* mRNA is upregulated in cervical cancer.** **A)** *USP13* mRNA expression from the GEO databases GSE6791, GSE7803, GSE9750 and GSE63514. Error bars represent the mean ± standard deviation. * p<0.5; ** p<0.01; *** p<0.001 (Student’s t-test).

**Supplementary Figure 3. HPV does not induce USP13 expression in cervical cancer cells. A)** RT-qPCR analysis of *USP13* mRNA expression in C33A cells transiently transfected with GFP or GFP tagged HPV18 E6, E7 or E6/E7. **B)** Representative western blot of C33A cells transiently transfected with GFP or GFP tagged HPV18 E6, E7 and E6/E7 and analysed for USP13 expression. Expression of HPV oncoproteins was confirmed by western blot for GFP, HPV18 E6 and HPV18 E7 expression using specific antibodies. GAPDH served as a loading control. Data shown is representative of at least three independent experiments. **C)** RT-qPCR analysis of *USP13* mRNA expression in HeLa cells transfected with a pool of specific siRNAs against HPV18 E6/E7 and analysed for phosphorylated USP13 expression. **D)** Representative western blot of HeLa cells transfected with a pool of specific siRNAs against HPV18 E6/E7 and analysed for phosphorylated USP13 expression. GAPDH served as a loading control. Data shown is representative of at least three independent experiments. Error bars represent the mean +/- standard deviation of a minimum of three biological repeats. *P<0.05, **P<0.01, ***P<0.001 (Student’s t-test). **E)** *USP13* expression in HPV+ and HPV- cervical cancer taken from the TCGA cervical cancer database. Error bars represent the mean +/- standard deviation. *P<0.05, **P<0.01, ***P<0.001 (Student’s t-test).

**Supplementary Figure 4. Depletion of USP13 has minimal impact on the proliferation of HPV- cervical cancer cells.** **A)** Representative western blot of C33A cells after transfection of a pool of four specific USP13 siRNA for 72 hours. Lysates were analysed for the expression of USP13 and GAPDH was used as a loading control. Data shown is representative of at least three independent experiments. **B)** Growth curve analysis of C33A cells after transfection of a pool of four specific USP13 siRNA for 72 hours. **C)** Colony formation assay (anchorage dependent growth) of C33A cells after transfection of a pool of four specific USP13 siRNA for 72 hours. **D)** Soft agar assay of C33A cells after transfection of a pool of four specific USP13 siRNA for 72 hours. Bars are the means ± standard deviation from at least three biological repeats. * p<0.5; ** p<0.01; *** p<0.001 (Student’s t-test).

**Supplementary Figure 5. USP13 does not regulate Mcl-1 expression in HPV- C33A cells.** **A)** Representative western blot of C33A cells after transfection of a pool of four specific USP13 siRNA for 72 hours. Lysates were analysed for the expression of USP13 and Mcl-1. GAPDH was used as a loading control. **B)** Representative western blot of HeLa and SiHa cells after transfection of FLAG-USP13 or FLAG-USP13 (C345A). Lysates were analysed for the expression of USP13 and Mcl-1. GAPDH was used as a loading control. Data shown is representative of at least three independent experiments.

**Supplementary Figure 6. Catalytically inactive USP13 interacts with Mcl-1.** **A)** HEK293T cells were transfected with Mcl-1, Flag-USP13 (C345A), or both Mcl-1 and FLAG-USP13 (C345A). Cells were treated with 10 µM MG132 for 6 hours and either Mcl-1 or USP13 were immunoprecipitated using an anti-Mcl1 or anti-FLAG antibody. Co-immunoprecipitated Mcl-1 or FLAG-USP13 were detected using the respective antibodies. GAPDH was used as a loading control. Data shown is representative of at least three independent experiments.

**Supplementary Figure 7. Spautin-1 does not sensitise HPV- C33A cells to ABT-263 treatment.** **A)** Cell viability assay (MTT) of C33A cells treated with increasing doses of ABT-263, with or without Spautin-1 (10 μM), for 24 hours. Bars are the means ± standard deviation from at least three biological repeats. * p<0.5; ** p<0.01; *** p<0.001 (Student’s t-test).
