# Supplementary figures and images for: The deubiquitinase (DUB) USP13 promotes Mcl-1 stabilisation in cervical cancer
Source: Oncogene. 2021 Feb 24;40(11):2112–29. doi: 10.1038/s41388-021-01679-8 (PMC7979541; doi:10.1038/s41388-021-01679-8)

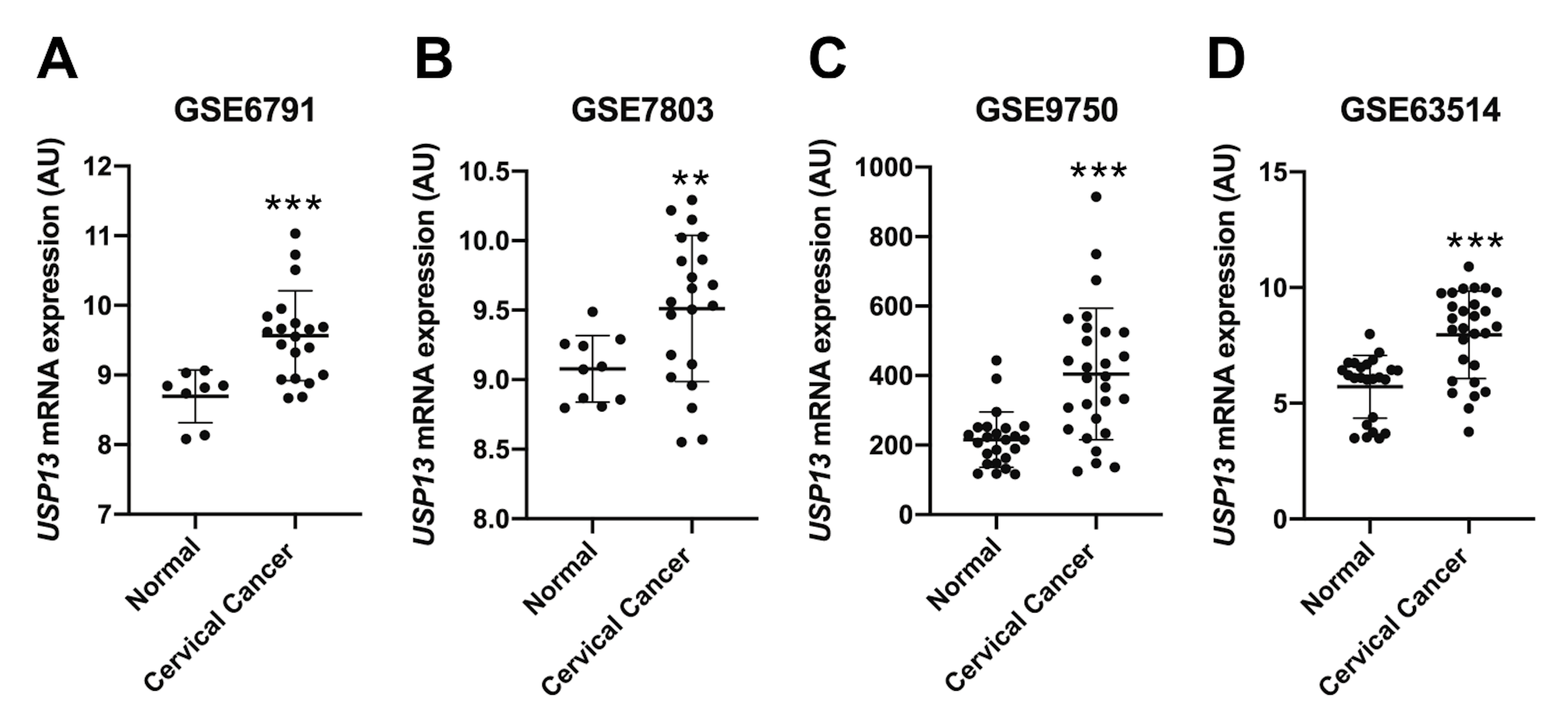

Supplement: Supplementary file 3 — Supp Fig 2 [file 41388_2021_1679_MOESM3_ESM.tif]

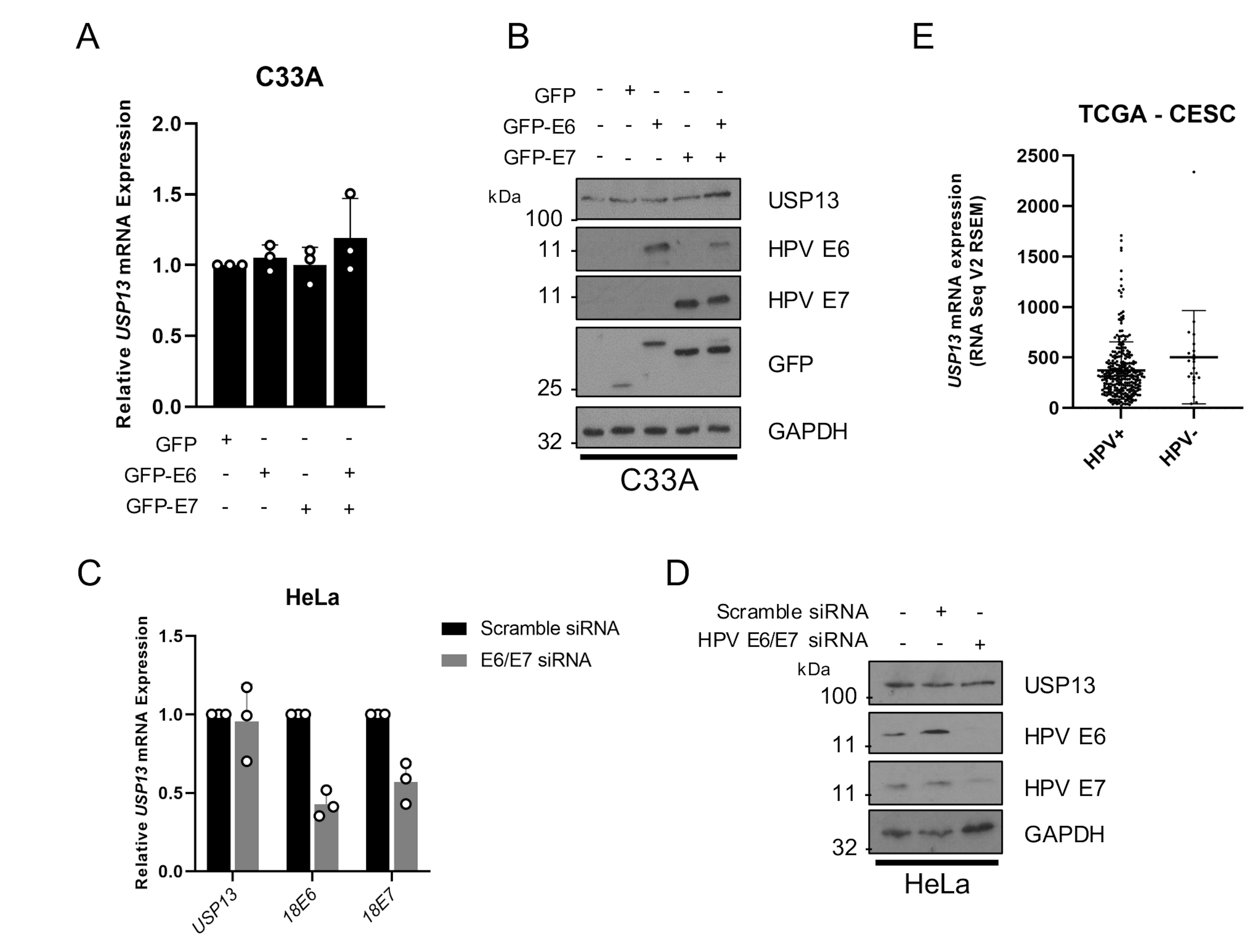

Supplement: Supplementary file 4 — Supp Fig 3 [file 41388_2021_1679_MOESM4_ESM.tif]

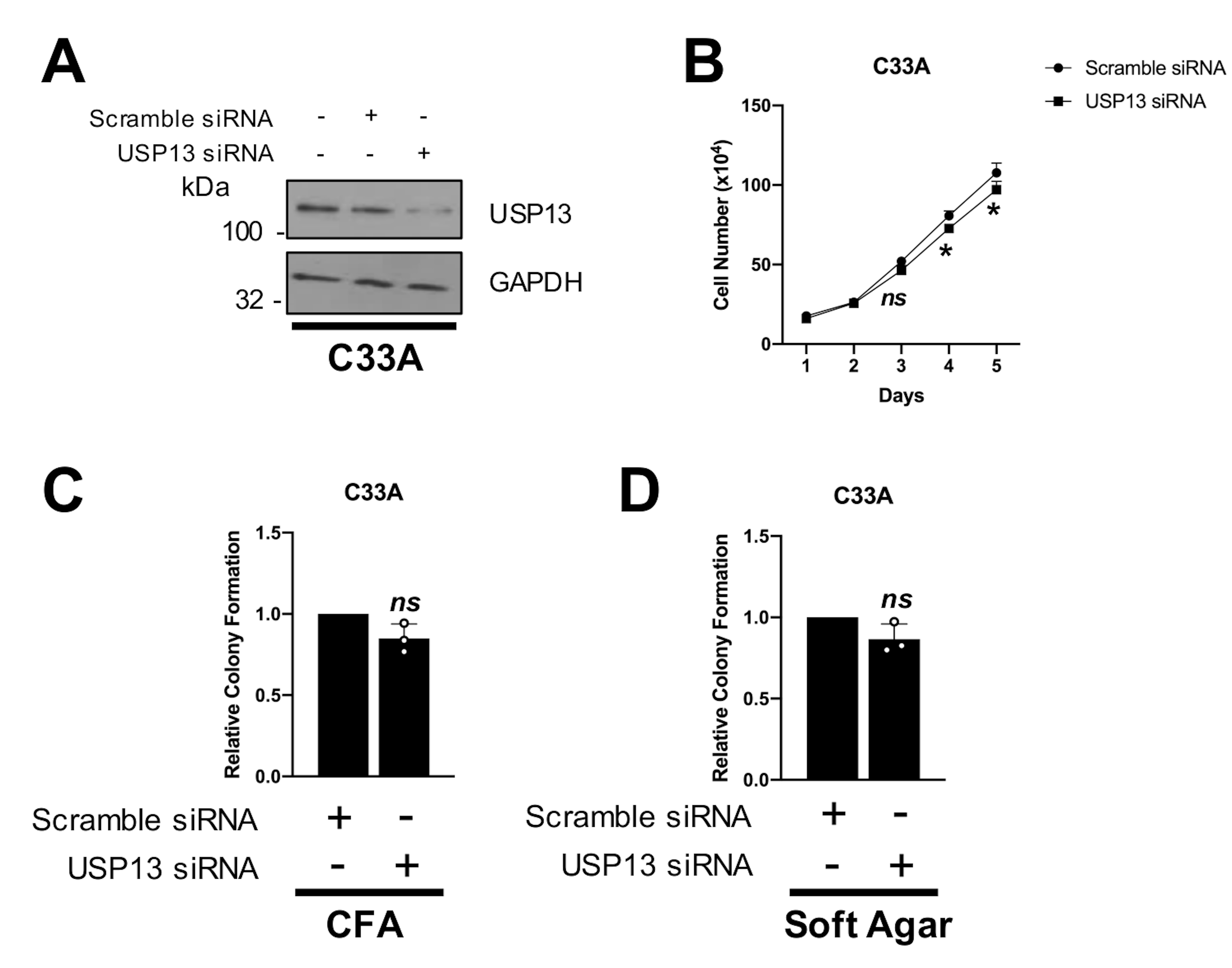

Supplement: Supplementary file 5 — Supp Fig 4 [file 41388_2021_1679_MOESM5_ESM.tif]

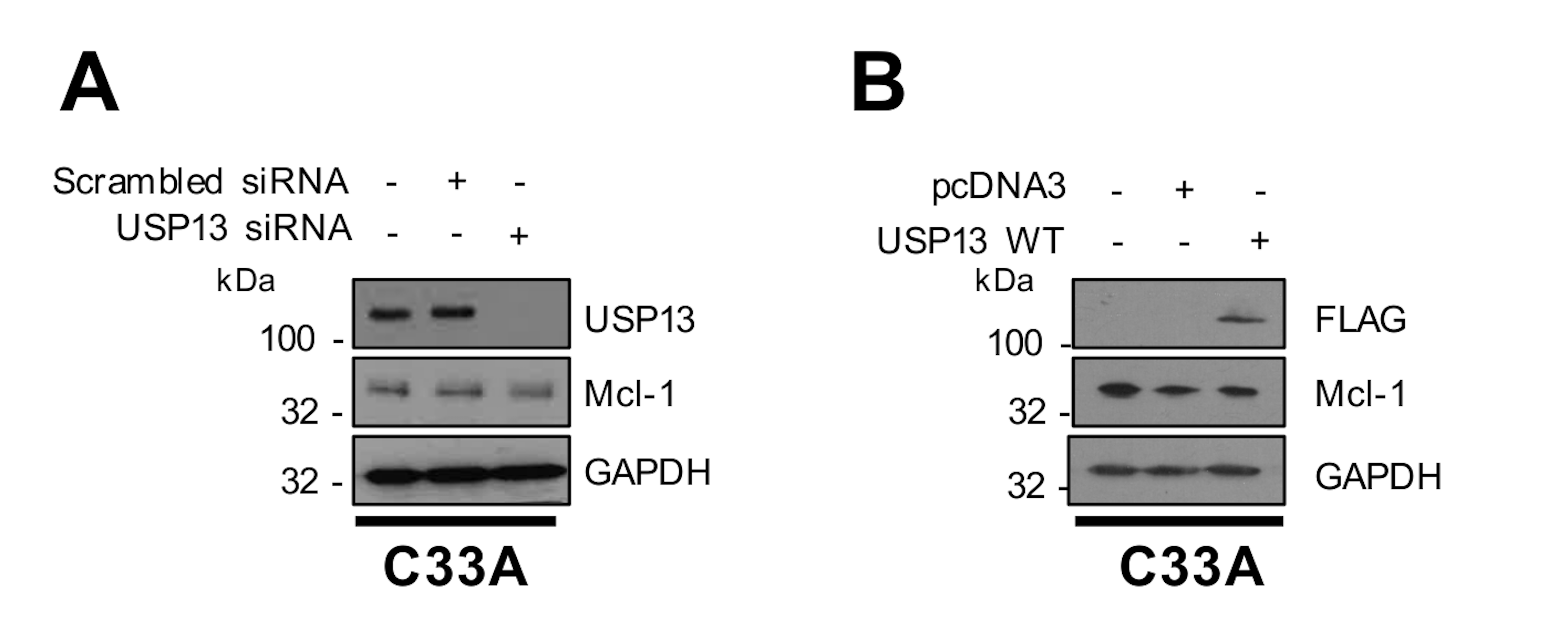

Supplement: Supplementary file 6 — Supp Fig 5 [file 41388_2021_1679_MOESM6_ESM.tif]

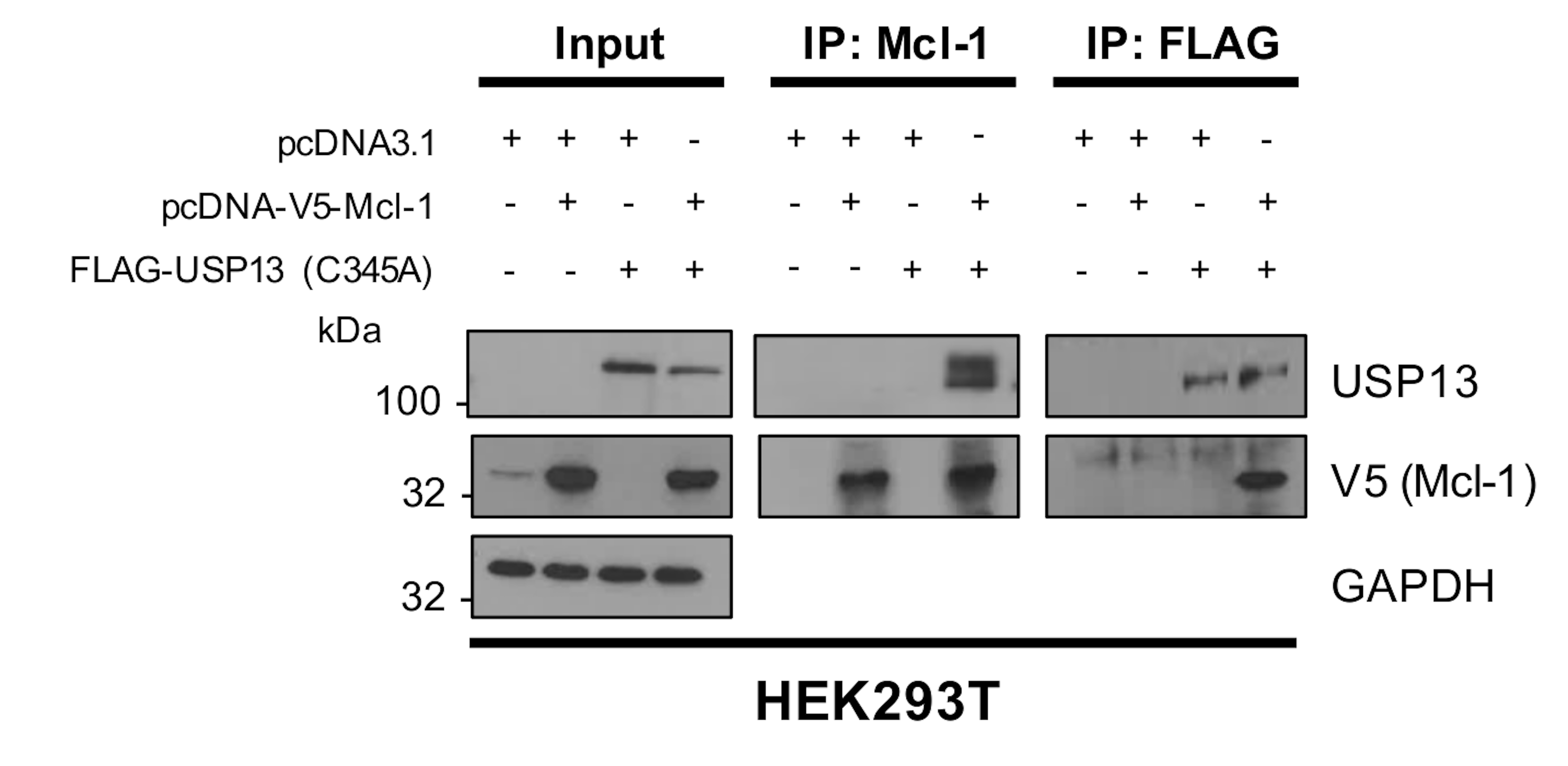

Supplement: Supplementary file 7 — Supp Fig 6 [file 41388_2021_1679_MOESM7_ESM.tif]

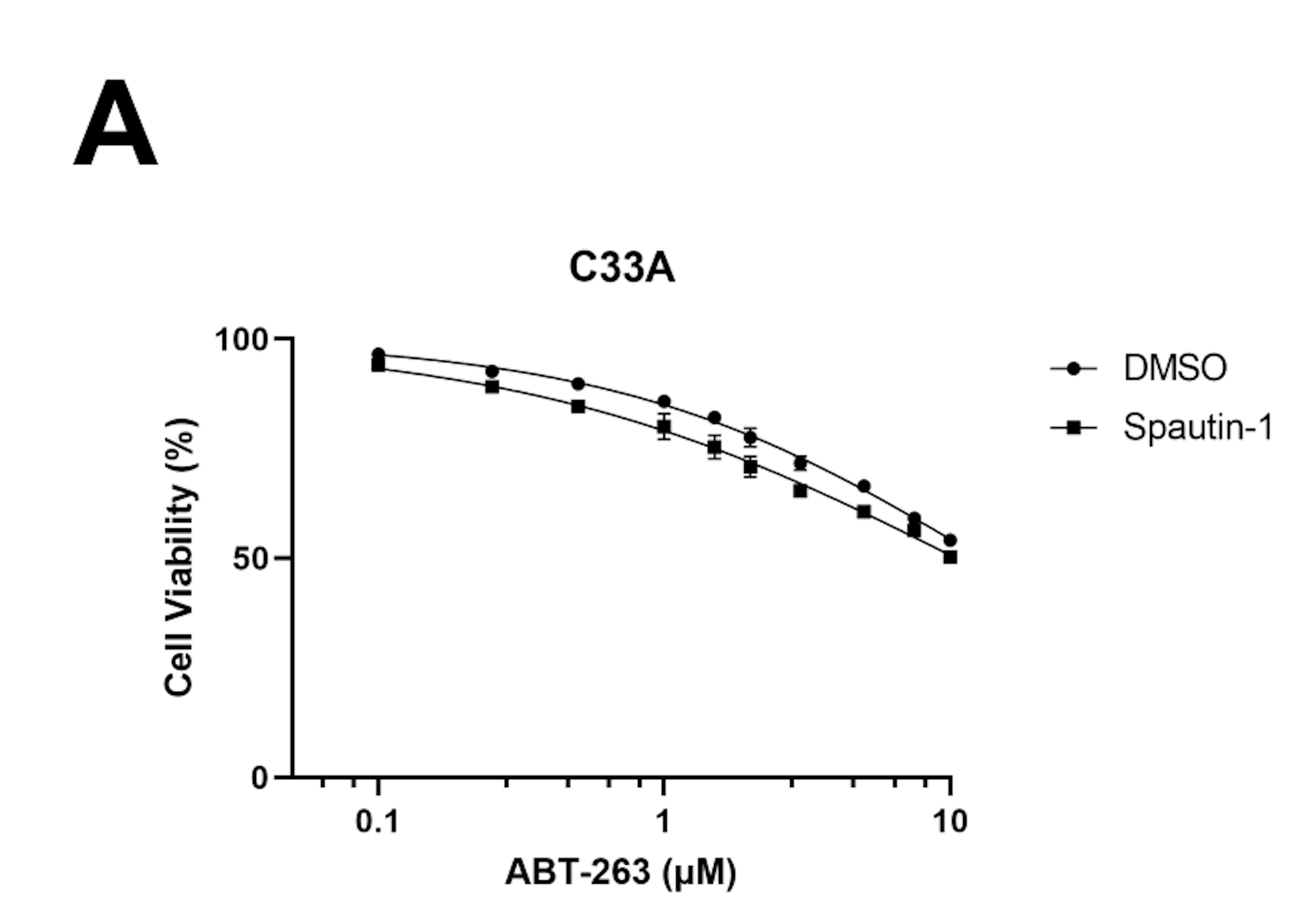

Supplement: Supplementary file 8 — Supp Fig 7 [file 41388_2021_1679_MOESM8_ESM.tif]
